# Comparative Evaluation of the Accuracy of Gingival Thickness Measurement by Clinical Evaluation and Intraoral Ultrasonography

**DOI:** 10.3390/jcm12134395

**Published:** 2023-06-29

**Authors:** Parisa Soltani, Jaber Yaghini, Kosar Rafiei, Mojdeh Mehdizadeh, Niccolò Giuseppe Armogida, Luigi Esposito, Gianrico Spagnuolo

**Affiliations:** 1 Department of Oral and Maxillofacial Radiology, Dental Implants Research Center, Dental Research Institute, School of Dentistry, Isfahan University of Medical Sciences, Isfahan 81745-33871, Iran; p.soltani@dnt.mui.ac.ir; 2 Department of Neurosciences, Reproductive and Odontostomatological Sciences, University of Naples “Federico II”, 80131 Naples, Italy; ng.armogida@gmail.com (N.G.A.); luigi.esposito0995@gmail.com (L.E.); 3 Department of Periodontics, Dental Implants Research Center, Dental Research Institute, School of Dentistry, Isfahan University of Medical Sciences, Isfahan 81745-33871, Iran; j_yaghini@dnt.mui.ac.ir; 4 Student Research Committee, School of Dentistry, Isfahan University of Medical Sciences, Isfahan 81745-33871, Iran; rafie_k@yahoo.com

**Keywords:** gingiva, periodontium, ultrasonography

## Abstract

This study aimed to investigate the accuracy of gingival thickness measurement by two methods of clinical evaluation and intraoral ultrasonography. The gingival thickness was measured in the midbuccal area of the right maxillary lateral incisor and first molar teeth in 30 individuals. For clinical measurement, a #15 K-file with rubber stops was vertically inserted 2 mm apical to the gingival margin and the length of the file in the tissue was measured using a digital caliper. Ultrasonographic measurement was performed using an intraoral probe on the gingival surface in the midbuccal area, at the entry point of the file. Statistical analysis was performed by paired t-test, correlation coefficient, and receiver operating characteristic (ROC) curve (α = 0.05). In the anterior region, the mean gingival thicknesses using ultrasonography (1.517 ± 0.293 mm) and clinical evaluation (1.610 ± 0.272 mm) were not significantly different (*p* = 0.434). In the posterior region, the mean gingival thicknesses were significantly different between ultrasonography (1.372 ± 0.442 mm) and clinical evaluation (1.626 ± 0.310 mm) (*p* = 0.006). The area under ROC curve values for ultrasonographic measurements in the anterior and posterior regions were 0.681 and 0.597, respectively. The use of ultrasonography with an intraoral probe has acceptable accuracy for the determination of gingival thickness, especially for the anterior regions.

## 1. Introduction

Olsson and Lindhe have suggested periodontal biotypes as parameters for classifying different morphological characteristics of the human periodontium [1]. Thin and scallop periodontal biotypes, which are accompanied by lower gingival thickness and width of the keratinized gingiva, are generally seen in relation with conical dentition with small interproximal contact areas. In contrast, thick and flat biotypes with thicker gingiva and higher width of the keratinized gingiva are more commonly seen in dentition with wider and shorter crowns and higher interproximal contact areas [2,3]. Gingival thickness is an important component of gingival biotypes and plays a considerable role in the outcome of regenerative periodontal and implant treatments [4,5,6,7]. In addition to periodontal and implant procedures, gingival thickness also potentially affects the results of orthodontic and restorative treatments [8,9]. Gingival thickness is dependent on genetic factors, age, sex, and oral hygiene and habits [3]. The risk of gingival recession, bone dehiscence, and alveolar ridge resorption after dental extraction is higher in thin gingival biotypes, while thick gingival biotypes have a higher incidence of periodontal pockets and fibrotic tissue response, as well as a more predictable outcome after regenerative treatments [10]. The gingival biotype can also affect the esthetic appearance, particularly in the maxillary and mandibular anterior teeth [11]. As a result, determination of gingival thickness prior to periodontal and implant surgeries as well as restorative treatments can be a crucial part of preoperative examinations. 

The measurement of gingival thickness is performed using various invasive and noninvasive methods. Invasive methods include histopathological examination and transgingival probing [12]. Ultrasonography, cone beam computed tomography (CBCT), and computed tomography (CT) are among the noninvasive techniques for the determination of gingival thickness [13,14,15]. The application of X-ray-based imaging modalities solely for the purpose of measuring gingival thickness can expose the patients to unnecessary radiation [16]. Therefore, ultrasonographic measurement of gingival thickness is gaining popularity. In fact, ultrasonography has been used in the dental practice due to its potential for developing real-time images, not using ionizing radiation, and its non-invasiveness [17]. 

Ultrasonography or ultrasound imaging is a modality that applies ultrasound waves, i.e., longitudinal mechanical sound waves with a frequency beyond the upper limit of human hearing (20 Hz to 20 KHz). Medical ultrasonography typically uses waves with a frequency ranging between 3 and 10 MHz, while high-frequency ultrasonography applies waves with a shorter wavelength and higher frequency (more than 10 MHz) compared to the conventional medical ultrasonography. High-frequency ultrasound waves are absorbed more easily by the tissues and therefore do not possess the ability for deep penetration. Therefore, although their application for the evaluation of deeper organs is limited, they are valuable for creating a high-resolution ultrasonographic image of superficial tissues. As a result, high-frequency ultrasonography is the ideal ultrasonographic imaging option in the field of dentistry [18]. The basic mechanisms that make ultrasonographic imaging possible can be described by the way the ultrasound waves interact with the tissues they encounter. Acoustic impedance is a term used to define the resistance of different tissues. This feature is highly dependent on the density of the tissue. In solid and dense materials, the ultrasound waves are reflected more, creating a hyperechoic bright image. Fluids, in contrast, mostly transmit the soundwave rather than reflecting it. Therefore, they create a hypoechoic darker image in ultrasound imaging. Air is a strong ultrasound wave reflector which makes the visualization of structures difficult.

Medical ultrasonography is generally categorized into amplitude modulation (A-mode) or brightness modulation (B-mode). A-mode ultrasonography results in one-dimensional wave-form images with spikes or peaks at the interface of different tissues. B-mode ultrasonography provides two-dimensional images of tissues with high resolution. B-mode ultrasonography, which is more common in medical imaging, is more expensive and requires more technical experience to obtain and interpret images [19]. During the recent years, intraoral ultrasonography has been used for various purposes including gingival and periodontal evaluation, assessment of periapical lesions, and characterization of benign and malignant neoplasms among other applications [20,21].

Several studies have been performed on the measurement of gingival thickness in individuals [22,23,24]. Some studies have used CBCT for this purpose. Sönmez et al. compared measurements of gingival thickness in 40 individuals using transgingival probing, CBCT, and ultrasonography. They reported an acceptable performance for ultrasonographic measurement, while CBCT measurement was challenged by low contrast resolution, resulting in lack of radiographic differentiation between the labial mucosa and the underlying gingiva [25]. Silva et al. used CBCT with and without lip retraction to measure the gingival thickness of anterior maxillary teeth. They reported that using lip retractors during CBCT scan acquisition allows precise measurement of gingival thickness by creating a distance between the labial mucosa and the anterior gingiva [26]. Moreover, Wang et al. in their systematic review reported that CBCT is a reliable tool for the measurement of the gingival thickness in anterior and posterior locations of the maxilla and mandible, while pointing out the need for retraction of buccal and labial tissues during CBCT imaging [27]. In any case, application of CBCT for gingival thickness measurement must be limited to cases that require three-dimensional imaging for other purposes, such as presurgical evaluation for implant treatments [16]. Therefore, some studies attempted to measure the gingival thickness using ultrasonographic evaluation. For instance, Savitha et al. reported that application of ultrasonography allows for quick, noninvasive, and precise measurement of gingival thickness [28]. However, intraoral probes have been used only in a few studies. This study aimed to evaluate the accuracy of ultrasonographic measurement of gingival thickness using an intraoral probe. Our hypothesis is that ultrasonographic measurement leads to accurate measurement of gingival thickness in both anterior and posterior regions.

## 2. Materials and Methods

All procedures followed were in accordance with the principles stated in the Declaration of Helsinki “Ethical Principles for Medical Research Involving Human Subjects”, adopted by the 18th World Medical Assembly, Helsinki, Finland, June 1964, and as amended most recently by the 64th World Medical Assembly, Fortaleza, Brazil, October 2013. The aim and the procedures of the study were fully explained to the participants and written informed consent was obtained from them. The participants were free to leave the study at any time. The protocol of this study was approved by the Ethics Committee at the Isfahan University of Medical Sciences (IR.MUI.RESEARCH.REC.1401.173). 

### 2.1. Sample Size and Patient Recruitment

The sample size was calculated based on the findings of the study by Sharma et al. [12]. The following formula was used, considering a significance level of 5% (α = 0.05) and power of 80% (β = 0.2) for detection of a difference up to 77% of the standard deviation (δ = 0.77σ):

$$n\geq \frac{{{2\;\sigma }^{2}\left(z_{1-\alpha /2}+z_{1-\beta }\right)}^{2}}{\delta^{2}}=\frac{2\;(1.96+0.84{)}^{2}}{{\left(0.77\right)}^{2}}=26.4≅27$$

The sample was selected among the individuals visiting the Department of Periodontics, Isfahan School of Dentistry, Iran, from July to November 2022. Inclusion criteria were systemically healthy individuals older than 18 years with healthy gingiva of the right maxillary lateral incisor and first molar and a pocket depth of less than 3 mm in these locations. Individuals who were unwilling to participate further, as well as pregnant and lactating women, smokers, and those taking medication affecting gingival tissues, such as phenytoin, calcium channel blockers, and cyclosporine, were excluded from the study. 

### 2.2. Measurement of Gingival Thickness

**Figure 1:**
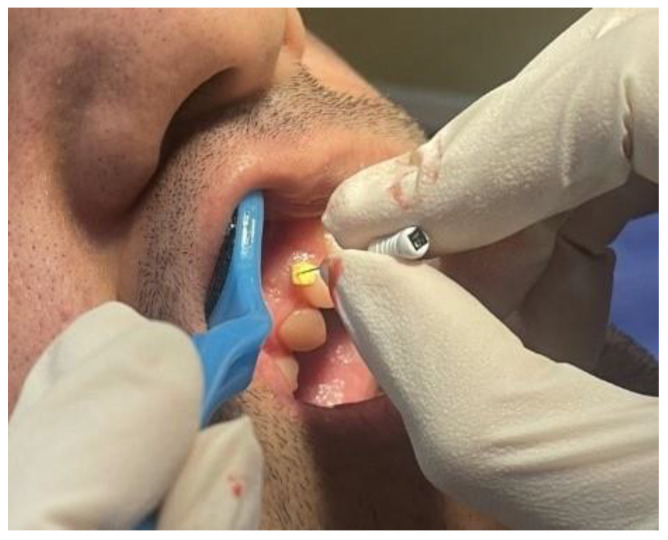
Measurement of gingival thickness using a hand K-file.

The transgingival clinical measurement was performed as the gold standard measurement technique [14,27]. First, anesthetic gel containing 5% lidocaine (Xylonor, Septodont, Maidstone, UK) was applied using a sterile cotton swab. Then, after a 1 min delay, a #15 hand K-file (Mani, Utsunomiya, Japan) equipped with rubber stops was inserted into the gingiva at 2 mm apical to the midbuccal region of right maxillary lateral incisor (anterior region) and first molar (posterior region). The rubber stops were fixed on the gingival surface (Figure 1) and the file was retrieved from the gingiva. Afterward, the gingival thickness was obtained by measurement of the length of the file tip to the rubber stop using a digital caliper (Pinrui DRO, Yongkang, Zhejiang, China). 

**Figure 2:**
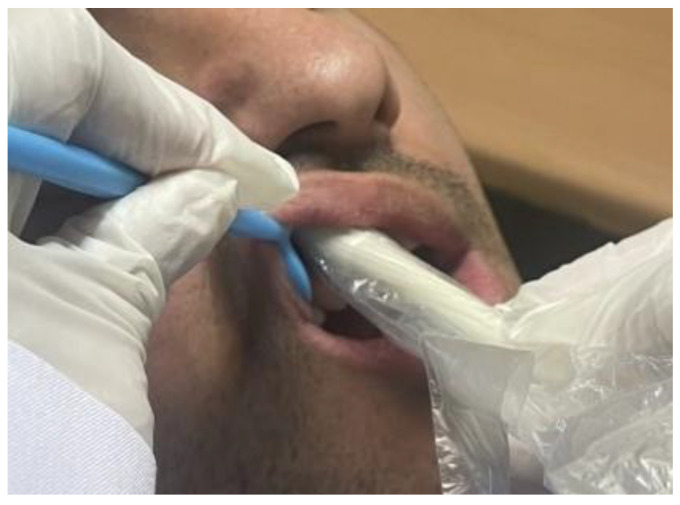
Measurement of gingival thickness using an intraoral ultrasonographic transducer.

B-mode ultrasonographic measurement of gingival thickness was performed using a 15 MHz intraoral probe in (Ecube 7, Alpinion Medical Systems, Seoul, Republic of Korea) using ultrasound transmission gel (Aquasonic 100, Parker Laboratories Inc., Almelo, The Netherlands) as a coupling agent. The probe was longitudinally placed on the midbuccal region of the right maxillary lateral incisor and first molar at the same position of the file entry point. The gingival thickness measurement was performed using a linear measurement tool (Figure 2).

### 2.3. Statistical Analysis

The data were entered into Statistical Package for the Social Sciences (SPSS, version 26, IBM Statistics, Armonk, NY, USA). First, the Shapiro–Wilk test was used to evaluate the distribution of the data. Due to the presence of a normal distribution of the data, independent *T*-test and paired T-test were used for further analysis (α = 0.05). Additionally, a receiver operating characteristic (ROC) curve was plotted and the area under curve (AUC), sensitivity, and specificity values of the ultrasonographic measurement were calculated [29]. The data are presented as means and standard deviations.

## 3. Results

A total of 30 individuals (15 males and 15 females) with an age range of 19 to 65 (mean 28.2 ± 9.5) participated in this study.

The gingival thickness values in the anterior region (midbuccal of the right maxillary lateral incisor) using the two measurement techniques of ultrasonography and K-file were not significantly different (*p* = 0.434). However, in the posterior region, a significant difference was seen between the values obtained using the two different measurement methods (*p* = 0.006) (Table 1). 

**Figure 3:**
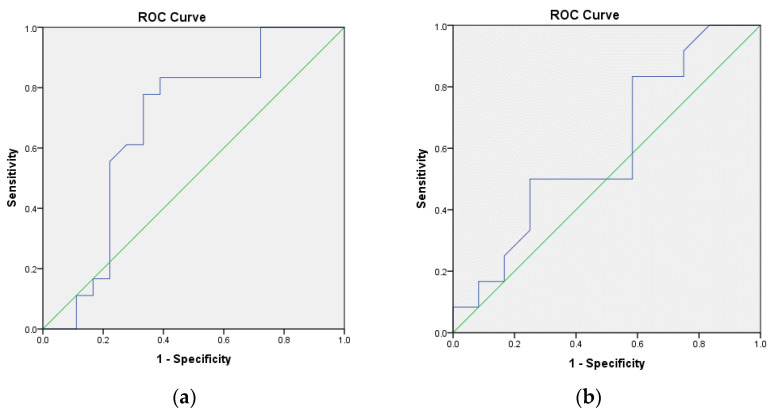
Receiver operative characteristic (ROC) curves for ultrasonographic measurement of the (**a**) anterior; (**b**) posterior regions.

The ROC curves for the ultrasonographic measurement of the gingival thickness in the anterior and posterior regions are shown in Figure 3. The AUC values for ultrasonographic measurement in the anterior and posterior regions were 0.681 and 0.597, respectively. While the sensitivity values for measurement of the gingival thickness in the anterior and posterior regions were equal (0.833), the specificity of measurement in the anterior region (0.611) was slightly higher than the posterior region (0.583).

## 4. Discussion

Based on the findings of this study, ultrasonographic measurement of gingival thickness showed more accuracy in the anterior region compared to the posterior region. In the anterior region, gingival thickness values obtained by ultrasonography showed no significant difference with the gold standard direct transgingival measurement. However, a significant difference existed between gingival thickness values measured by the two methods in the posterior region. In general, ultrasonographic measurement tended to underestimate the gingival thickness compared to the gold standard transgingival measurement. 

Prediction of the success and outcome of treatments before initiation of the therapeutic phase is an integral part of treatment planning and risk prognosis. Thus, determination and evaluation of the factors that can affect the outcome of individual treatments is of utmost importance. Gingival thickness plays an important role in the process of gingival recession, wound healing, flap design in regenerative surgeries, implant surgeries, and root coverage procedures [30,31,32,33]. Additionally, the gingival condition can be altered as a result of dental treatments and procedures [34,35].

Some studies have used CBCT for determination of gingival thickness. For instance, Sönmez et al. compared the accuracy of gingival thickness measurements in 40 individuals who were candidates for implant insertion, using transgingival probing, CBCT, and ultrasonography. They reported acceptable performance of ultrasonographic measurement, while CBCT measurements were not compatible with the transgingival ones. The authors concluded that due to the low contrast resolution in CBCT imaging, radiographic differentiation between the labial mucosa and the underlying gingiva is challenging [25]. Supporting this conclusion, Silva et al. reported that using lip retractors during CBCT scan acquisition allows precise measurement of gingival thickness by creating a distance between the labial mucosa and the anterior gingiva [26]. Additionally, in their systematic review in in 2022, Wang et al. reported that CBCT is a reliable tool for measuring the gingival thickness in different location of the oral cavity [27]. Nevertheless, application of CBCT for gingival thickness measurement must be limited to cases that require three-dimensional imaging for other purposes, such as presurgical evaluation for implant treatments [16]. 

Ultrasonography has gained popularity in dental practice due to its potential for developing real-time images, not using ionizing radiation, and its non-invasiveness [17]. Ultrasonography or ultrasound imaging uses ultrasound waves with a frequency beyond the limit of human hearing. Ultrasound waves are mechanical longitudinal waves generated from piezoelectric materials in the ultrasonographic transducer and can propagate in different media. High-frequency ultrasonography applies waves with a shorter wave-length and higher frequency (more than 10 MHz) compared to the conventional medical ultrasonography. High-frequency ultrasound waves are absorbed more easily by the tissues and thus are not as penetrating. Therefore, they are best applied for the imaging of superficial structures and are not suitable for evaluating deeper organs. The characteristics and features of high-frequency ultrasonography make it the optimal ultrasonographic imaging option in the field of dentistry [18]. The basic mechanisms that make ultrasonographic imaging possible can be described by the way the ultrasound waves interact with the tissues they encounter. Acoustic impedance is a term used to define the resistance of different tissues. This feature is highly dependent on the density of the tissue. In solid and dense materials, the ultrasound waves are reflected more, creating a hyperechoic bright image. Fluids, in contrast, mostly transmit the soundwave rather than reflecting it. Therefore, they create a hypoechoic darker image in ultrasound imaging. Air is a strong ultrasound wave reflector which makes the visualization of structures difficult.

In recent years, intraoral ultrasonography has been used for various purposes including gingival and periodontal evaluation, assessment of periapical lesions, and characterization of benign and malignant neoplasms among other applications [20,21]. Instead of transgingival probing, which requires the insertion of sterile periodontal probes into the gingiva, studies have advocated the use of noninvasive methods, including ultrasonography for the measurement of gingival thickness. Ultrasound waves pass through soft tissue but are not able to traverse bone, resulting in an anechoic appearance below the bone. However, the bone surface appears hyperechoic as a result of the significant acoustic mismatch between the superficial soft tissue and underlying bony structures. Therefore, gingival thickness can be measured using ultrasonography by measurement of the tissue superficial to the hyperechoic bone surface. In several studies, the measurement of gingival thickness was performed using extraoral ultrasonographic probes. For instance, Savitha et al. attempted ultrasonographic measurements of the gingival thickness using extraoral probes. Although they reported that ultrasonography can be considered as a quick, noninvasive, and precise technique for the measurement of gingival thickness, the use of such large extraoral probes are limited intraorally [28]. Recently smaller and more practical intraoral probes have been available for various oral diagnostic applications. Due to the novelty of these probes, the number of studies comparing the accuracy of measurements using these probes with clinical measurements is scarce. 

In 2012, Salmon et al. developed an intraoral ultrasonographic probe to measure several variables in the oral cavity. Using their 20 MHz intraoral probe, they were able to visualize the periodontal structures and perform measurements with good inter-observer agreement. However, they did not used a gold standard for their measurements. In 2015, Slak et al. performed a pilot study on the accuracy of the measurement of gingival thickness made by a 50 MHz intraoral ultrasonographic probe on a porcine model and reported good agreement with the invasive methods [36].

In 2015, Borges et al. performed measurements of gingival thickness on 29 patients prior to gingivectomy. They employed an intraoral probe (frequency not mentioned) to perform gingival measurements in different regions of the oral cavity. They reported significant differences between measurements in the incisor and canine region but not the posterior region [37]. These findings are not consistent with our findings. The reason can be attributed to different specifications and application of ultrasonographic probes. Moreover, Tattan et al. in 2020 evaluated periodontal structures using a 24 MHz intraoral ultrasonographic probe. They concluded that intraoral ultrasonographic measurements have a relatively good correlation with direct clinical measurements [38]. However, they did not specify their measurements and findings based on the location. Their findings are consistent with our observations in the anterior region, indicating good agreement between the clinical invasive and ultrasonographic measurements of the gingival thickness. In a study performed by Kloukos et al. in 2018, it was reported that ultrasonographic measurement of gingival thickness yields results statistically similar to those using transgingival probing, although ultrasonographic measurements were slightly higher [39]. Meanwhile, we observed that the mean values for ultrasonographic evaluation were generally lower than those of clinical transgingival measurements. In the study by Kluokos, it is mentioned that a 5 MHz transducer was used. This frequency is lower than the frequency required to visualize the periodontal structures. A systematic review of the literature performed by Wang et al. in 2022 focused on different methods for the measurement of gingival thickness in different intraoral regions. The authors recommended ultrasonography as a useful and relatively reliable option for the measurement of gingival thickness compared with direct probing for the anterior regions of the gingiva in the maxilla and mandible. However, in line with our findings, it was mentioned that a significant difference existed between the gingival thickness values measured by ultrasonography and direct gold standard probing [27]. The reason for this finding can be associated with the difficulty of appropriate placement of the ultrasonographic probe in the posterior area due to its diameter and profile. A recent systematic review and meta-analysis by Fan et al. in 2023 revealed that ultrasonography can be considered as a reliable tool for measurement of the gingival thickness in patients. However, the authors recommended conducting further standardized clinical studies with larger sample sizes due to the very low certainty of the results in the meta-analysis [40]. 

Medical ultrasonography is classified as either amplitude modulation (A-mode) or brightness modulation (B-mode). A-mode ultrasonography provides one-dimensional wave-form images with spikes or peaks at the interface of different tissues, whereas B-mode ultrasound provides two-dimensional images with high resolution. B-mode ultrasonography, which is more common in medical imaging, is generally more expensive and requires more technical experience for its appropriate application [19]. Ultrasound in both modes can be used to measure gingival thickness ex vivo and in vivo. In the present study, B-mode ultrasonography was used, as it is the prevailing option for ultrasonography in the dental setting. However, A-mode ultrasonography is also useful in the measurement of gingival thickness by depicting peaks at the interface between the gingival tissue and bone. Thus, the interpretation and measurement using A-mode ultrasonography can be simpler and less costly.

As mentioned, the thickness measurements yielded by ultrasonography were generally lower than transgingival measurements. This finding can be a result of the slight pressure of the ultrasonographic transducer in the gingival tissue which can slightly compress the gingival tissue and provide lower thickness values. Transducer pressure on the soft tissue may cause a decrease in gingival thickness when measuring using ultrasonographic probes. Another finding in our study was the significant difference in ultrasonographic measurements with transgingival ones in the posterior region. Longitudinal placement of the hockey probe used in our study in the posterior region was more of a challenge. This can be considered as one of the limitations of our study. Similar observations were also found in other studies [41]. As mentioned, the size and profile of the ultrasonic transducer can make its placement in the posterior area more difficult. This can explain the smaller accuracy of ultrasonic device measurements in the posterior area [27]. The application of circular transducers can overcome this difficulty in appropriate positioning and adjustment of the probe within the gingiva. A comparison of the performance of different ultrasonographic transducers in measurements in different regions of the oral cavity is required in future investigations [36]. Therefore, performing accurate measurements in all regions of the oral cavity requires an appropriate type of ultrasonographic transducer, with a frequency higher than 15 MHz, as well as proficiency of the operator. 

## 5. Conclusions

The accuracy of ultrasonographic measurements of the gingival thickness in the anterior region was higher than that in the posterior region. Additionally, compared to the transgingival measurements, a significant difference was observed in measurements of the posterior region, but not the anterior region. Chairside ultrasonographic measurement of the gingival thickness can be performed before regenerative procedures, particularly in the anterior region.

## Figures and Tables

**Table 1:** Minimum, maximum, and mean (SD) values (mm) of gingival thickness measured by ultrasonography and transgingival measurement using K-file.

Region	Method	Minimum	Maximum	Mean (SD)	*p*-Value
Anterior	Ultrasonographic	1.08	1.98	1.517 (0.293)	0.434
	Transgingival	1.25	2.05	1.610 (0.272)	
Posterior	Ultrasonographic	0.81	2.20	1.372 (0.442)	0.006 *
	Transgingival	1.12	2.10	1.626 (0.310)	

* Indicates significant difference (*p* < 0.05).

## Data Availability

All data are contained within the article.

## References

[1] Olsson M., Lindhe J. (1991). Periodontal characteristics in individuals with varying form of the upper central incisors. J. Clin. Periodontol..

[2] Stellini E., Comuzzi L., Mazzocco F., Parente N., Gobbato L. (2013). Relationships between different tooth shapes and patient’s periodontal phenotype. J. Periodontal Res..

[3] Yin X.-J., Wei B.-Y., Ke X.-P., Zhang T., Jiang M.-Y., Luo X.-Y., Sun H.-Q. (2020). Correlation between clinical parameters of crown and gingival morphology of anterior teeth and periodontal biotypes. BMC Oral Health.

[4] Hwang D., Wang H.L. (2006). Flap thickness as a predictor of root coverage: A systematic review. J. Periodontol..

[5] Ganvir M.N., Parwani S.R., Chaudhary D.S., Parwani R., Dadlani H., Vikey A.K., Kawadkar K.P., Jaju N.S., Armogida N.G., Spagnuolo G. (2022). Comparative Evaluation of Azadirachta indica (Neem) Chip and Soft Tissue Diode Lasers as a Supplement to Phase I Periodontal Therapy in Localized Chronic Moderate Periodontitis: A Randomized Controlled Clinical Trial. Int. J. Dent..

[6] Cafiero C., Spagnuolo G., Marenzi G., Martuscelli R., Colamaio M., Leuci S. (2021). Predictive periodontitis: The most promising salivary biomarkers for early diagnosis of periodontitis. J. Clin. Med..

[7] Consiglio R., Rengo S., Liguoro D., Riccitiello F., Formisano S., Palumbo G., Di Jeso B. (1998). Inhibition by glass-ionomer cements of protein synthesis by human gingival fibroblasts in continuous culture. Arch. Oral. Biol..

[8] Boke F., Gazioglu C., Akkaya S., Akkaya M. (2014). Relationship between orthodontic treatment and gingival health: A retrospective study. Eur. J. Dent..

[9] Zawawi K.H., Al-Zahrani M.S. (2014). Gingival biotype in relation to incisors’ inclination and position. Saudi Med. J..

[10] Alkan Ö., Kaya Y., Alkan E.A., Keskin S., Cochran D.L. (2018). Assessment of gingival biotype and keratinized gingival width of maxillary anterior region in individuals with different types of malocclusion. Turk. J. Orthod..

[11] Humagain M., Rokaya D., Srii R., Dixit S., Kafle D. (2016). Gender Based Comparison of Gingival Zenith Esthetics. Kathmandu Univ. Med. J. (KUMJ).

[12] Kloukos D., Koukos G., Gkantidis N., Sculean A., Katsaros C., Stavropoulos A. (2021). Transgingival probing: A clinical gold standard for assessing gingival thickness. Quintessence Int..

[13] de Freitas Silva B.S., Silva J.K., Silva L.R., de Lima K.L., Mezaiko E., Roriz V.M., Evangelista K., Yamamoto-Silva F.P. (2023). Accuracy of cone-beam computed tomography in determining gingival thickness: A systematic review and meta-analysis. Clin. Oral Investig..

[14] El Khalifa M., Abu el Sadat S.M., Gaweesh Y.S., Gaweesh Y.Y. (2022). Assessment of Gingival Thickness Using CBCT Compared to Transgingival Probing and Its Correlation with Labial Bone Defects: A Cross-Sectional Study. Int. J. Oral Maxillofac. Implant..

[15] Renaud M., Delpierre A., Becquet H., Mahalli R., Savard G., Micheneau P., Carayon D., Denis F. (2023). Intraoral Ultrasonography for Periodontal Tissue Exploration: A Review. Diagnostics.

[16] Moore C.A., Law J.K., Retout M., Pham C.T., Chang K.C.J., Chen C., Jokerst J.V. (2022). High-resolution ultrasonography of gingival biomarkers for periodontal diagnosis in healthy and diseased subjects. Dentomaxillofacial Radiol..

[17] Di Stasio D., Romano A., Montella M., Contaldo M., Petruzzi M., Hasan I., Serpico R., Lucchese A. (2022). Quantitative Ultrasound Analysis of Oral Mucosa: An Observational Cross-Sectional Study. Appl. Sci..

[18] Reda R., Zanza A., Cicconetti A., Bhandi S., Miccoli G., Gambarini G., Di Nardo D. (2021). Ultrasound Imaging in Dentistry: A Literature Overview. J. Imaging.

[19] Wagner D.R., Thompson B.J., Anderson D.A., Schwartz S. (2019). A-mode and B-mode ultrasound measurement of fat thickness: A cadaver validation study. Eur. J. Clin. Nutr..

[20] Tanaka R., Lau K., Yeung A.W., Leung W.K., Hayashi T., Bornstein M.M., Tonetti M.S., Pelekos G. (2023). Diagnostic application of intraoral ultrasonography to assess furcation involvement in mandibular first molars. Dentomaxillofacial Radiol..

[21] Kawano S., Hattori T., Mikami Y., Chikui T., Kawazu T., Sakamoto T., Maruse Y., Tanaka S., Hamada E., Hiwatashi M. (2023). Prediction of nodal metastasis based on intraoral sonographic findings of the primary lesion in early-stage tongue cancer. Int. J. Oral Maxillofac. Surg..

[22] Sun M., Liu X., Xia T., Meng H. (2021). Non-invasive evaluation of labial gingival and alveolar crest thickness in the maxillary anterior teeth region by 15-MHz B-mode ultrasonography. BMC Oral Health.

[23] Chifor R., Badea A.F., Chifor I., Mitrea D.-A., Crisan M., Badea M.E. (2019). Periodontal evaluation using a non-invasive imaging method (ultrasonography). Med. Pharm. Rep..

[24] Sun M., Yao W., Deng Y.Q., Cao J., Meng H. (2020). Measurements of buccal gingival and alveolar crest thicknesses of premolars using a noninvasive method. Med. Ultrason..

[25] Sönmez G., Kamburoğlu K., Gülşahı A. (2021). Accuracy of high-resolution ultrasound (US) for gingival soft tissue thickness mesurement in edentulous patients prior to implant placement. Dentomaxillofacial Radiol..

[26] Silva J.N.N., de Andrade P.F., Sotto-Maior B.S., Assis N.M.S.P., Carvalho A.C.P., Devito K.L. (2017). Influence of lip retraction on the cone beam computed tomography assessment of bone and gingival tissues of the anterior maxilla. Oral Surg. Oral Med. Oral Pathol. Oral Radiol..

[27] Wang J., Cha S., Zhao Q., Bai D. (2022). Methods to assess tooth gingival thickness and diagnose gingival phenotypes: A systematic review. J. Esthet. Restor. Dent..

[28] Savitha B., Vandana K. (2005). Comparative assesment of gingival thickness using transgingival probing and ultrasonographic method. Indian J. Dent. Res..

[29] Del Giudice C., Vaia E., Liccardo D., Marzano F., Valletta A., Spagnuolo G., Ferrara N., Rengo C., Cannavo A., Rengo G. (2021). Infective Endocarditis: A Focus on Oral Microbiota. Microorganisms.

[30] Blasi G., Vilarrasa J., Abrahamian L., Monje A., Nart J., Pons R. (2023). Influence of immediate postoperative gingival thickness and gingival margin position on the outcomes of root coverage therapy: A 6 months prospective case series study using 3D digital measuring methods. J. Esthet. Restor. Dent..

[31] Mostafa D., Fatima N. (2022). Gingival recession and root coverage up to date, a literature review. Dent. Rev..

[32] Khorshed A., Vilarrasa J., Monje A., Nart J., Blasi G. (2023). Digital evaluation of facial peri-implant mucosal thickness and its impact on dental implant aesthetics. Clin. Oral Investig..

[33] Nisanci Yilmaz M.N., Koseoglu Secgin C., Ozemre M.O., Inonu E., Aslan S., Bulut S. (2022). Assessment of gingival thickness in the maxillary anterior region using different techniques. Clin. Oral Investig..

[34] Heboyan A., Manrikyan M., Zafar M.S., Rokaya D., Nushikyan R., Vardanyan I., Vardanyan A., Khurshid Z. (2021). Bacteriological Evaluation of Gingival Crevicular Fluid in Teeth Restored Using Fixed Dental Prostheses: An in Vivo Study. Int. J. Mol. Sci..

[35] Avetisyan A., Markaryan M., Rokaya D., Tovani-Palone M.R., Zafar M.S., Khurshid Z., Vardanyan A., Heboyan A. (2021). Characteristics of periodontal tissues in prosthetic treatment with fixed dental prostheses. Molecules.

[36] Slak B., Daabous A., Bednarz W., Strumban E., Maev R.G. (2015). Assessment of gingival thickness using an ultrasonic dental system prototype: A comparison to traditional methods. Ann. Anat.-Anat. Anz..

[37] Borges G.J., Ruiz L.F.N., de Alencar A.H.G., Porto O.C.L., Estrela C. (2015). Cone-beam computed tomography as a diagnostic method for determination of gingival thickness and distance between gingival margin and bone crest. Sci. World J..

[38] Tattan M., Sinjab K., Lee E., Arnett M., Oh T.J., Wang H.L., Chan H.L., Kripfgans O.D. (2020). Ultrasonography for chairside evaluation of periodontal structures: A pilot study. J. Periodontol..

[39] Kloukos D., Koukos G., Doulis I., Sculean A., Stavropoulos A., Katsaros C. (2018). Gingival thickness assessment at the mandibular incisors with four methods: A cross-sectional study. J. Periodontol..

[40] Fan S., Sáenz-Ravello G., Al-Nawas B., Schiegnitz E., Diaz L., Sagheb K. (2023). The feasibility of ultrasonography for the measurement of periodontal and peri-implant phenotype: A systematic review and meta-analysis. Clin. Implant. Dent. Relat. Res..

[41] Gürlek Ö., Sönmez Ş., Güneri P., Nizam N. (2018). A novel soft tissue thickness measuring method using cone beam computed tomography. J. Esthet. Restor. Dent..

